# Polarimetry of *Pinctada fucata* nacre indicates myostracal layer interrupts nacre structure

**DOI:** 10.1098/rsos.160893

**Published:** 2017-02-15

**Authors:** Rebecca A. Metzler, Joshua A. Jones, Anthony J. D'Addario, Enrique J. Galvez

**Affiliations:** Department of Physics and Astronomy, Colgate University, 13 Oak Drive, Hamilton, NY 13346, USA

**Keywords:** nacre, myostracal layer, imaging polarimetry, orientation

## Abstract

The inner layer of many bivalve and gastropod molluscs consists of iridescent nacre, a material that is structured like a brick wall with bricks consisting of crystalline aragonite and mortar of organic molecules. Myostracal layers formed during shell growth at the point of muscle attachment to the shell can be found interspersed within the nacre structure. Little has been done to examine the effect the myostracal layer has on subsequent nacre structure. Here we present data on the structure of the myostracal and nacre layers from a bivalve mollusc, *Pinctada fucata*. Scanning electron microscope imaging shows the myostracal layer consists of regular crystalline blocks. The nacre before the layer consists of tablets approximately 400 nm thick, while after the myostracal layer the tablets are approximately 500 nm thick. A new technique, imaging polarimetry, indicates that the aragonite crystals within the nacre following the myostracal layer have greater orientation uniformity than before the myostracal layer. The results presented here suggest a possible interaction between the myostracal layer and subsequent shell growth.

## 1. Introduction

The majority of marine bivalves and gastropods produce shells that consist of an outer calcite prismatic layer and an inner aragonite nacre layer. The nacreous layer has a unique multi-scale structure of ordered crystals that gives it an extraordinary ability to withstand fracture [1–7]. This structure also yields a beautiful iridescence caused by interference and diffraction of light [8,9]. Within the nacre structure are myostracal layers that form where muscles attach to the shell [10–12].

The basic nacre structure consists of a ‘brick and mortar’ configuration. The ‘mortar’ is organic and the ‘bricks’ are flat aragonite crystals that are typically approximately 10 µm long and approximately 0.5 µm thick [8,9,13]. Despite consisting of aggregated nanoparticles, the aragonite bricks in bivalves and gastropods diffract as single crystals [14–16]. In bivalves, the aragonite bricks are largely co-aligned, such that the crystals have their three crystallographic axes (*a*,*b*,*c*) parallel to one another [17–20]. In gastropods, the *c*-axes of the aragonite bricks are generally co-aligned, while the *a*- and *b*-axes are randomly oriented throughout the structure [9,18,21,22].

The organism connects to its protective shell through various muscles. Thin aragonitic shell layers with a prismatic structure form at these attachment points, which are called myostracal layers [11,12,23]. These myostracal layers are embedded within the nacre structure, resulting in there being nacre before and after the myostracal layer. While recent studies have explored the proteomic differences between the nacre and myostracum, the role the myostracum plays in subsequent nacre growth has not been examined [11,24].

Here we use a new technique, imaging polarimetry [25–29], to probe the myostracal layer of the bivalve *Pinctada fucata* and its interaction with nacre growth. Our technique [28,29] uses single-wavelength light of selectable polarization to probe the sample, examining the light transmitted through the sample as a function of polarization and position. Our polarimetry analysis, in conjunction with scanning electron microscopy (SEM), finds an increase in tablet thickness and a decrease in the variation in orientation of the nacre tablets from before the myostracal layer to after the myostracal layer.

## 2. Material and methods

**Figure 1.**
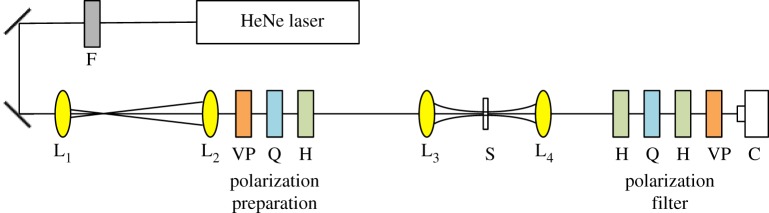
A diagram of the experimental set-up with the sample being denoted as S. The incoming light (λ = 632.8 nm) from the HeNe laser was sent through a filter (F) to adjust the intensity to a level appropriate for the camera, reflected off two mirrors (denoted by the black lines) and collimated by lenses (L_1_ and L_2_) before having polarization information encoded into the beam. The polarization state of the incoming beam was selected by sending the light through a vertical polarizer (VP) to ensure that the beam has a uniform polarization, followed by a quarter-wave plate (Q) and a half-wave plate (H) which encode the polarization of interest. Following polarization encoding, the beam was focused onto the sample (lens L_3_). The light transmitted through the sample was recollimated by lens L_4_ and sent through a polarization filter (half-wave plate (H), quarter-wave plate (Q), half-wave plate (H) and vertical polarizer (VP)), where the polarization information was decoded before arriving at the camera (C).

Figure 1 presents a diagram of the experimental set-up used in the polarimetry experiments [28,29]. A helium–neon (HeNe) laser beam of wavelength 632.8 nm was passed through a neutral density filter (*F*) to adjust the intensity to a level that was appropriate for the camera. The polarization state of the incoming light was selected by passing the light through a vertical polarizer (VP) to ensure that all of the incoming light was of a uniform polarization. The polarizer was followed by a quarter-wave plate (QWP) and half-wave plate (HWP), which were adjusted to give the light one of the desired polarization states: linear vertical (V), linear horizontal (H), linear anti-diagonal (A; −45° to horizontal), linear diagonal (D; +45° to horizontal), left circular (L) and right circular (R). The light was then focused onto the thin sample (S) using a 20× microscope objective. With the input light having a spot size of 0.45 mm, we estimate that the illuminated region, at the sample, had a diameter of about 15 µm. The transmitted light was imaged onto a digital camera (C) with a 10× objective. Before the camera there were a series of optical elements (HWP, QWP, HWP and VP) serving as a polarization filter [30]. For each incoming polarization, the polarization filter was adjusted six times to detect light in each of the six possible polarization states mentioned above, and an image was collected for each case. The images were then combined to produce a composite image that had both amplitude and polarization information for the illuminated portion of the sample. This consisted of extracting the Stokes parameters of each imaged point [26]:

2.1 $$\begin{array}{rl} s_{0} & =I_{H}+I_{V}, \end{array}$$

2.2 $$\begin{array}{rl} s_{1} & =I_{H}-I_{V}, \end{array}$$

2.3 $$\begin{array}{rl} s_{2} & =I_{D}-I_{A} \end{array}$$

2.4 $$\begin{array}{rl} \;\text{and}\;\;s_{3} & =I_{R}-I_{L}, \end{array}$$

with *I_i_* being the recorded intensity with filter *i*. The state of polarization of the light is completely determined by these four parameters. If a sample were completely uniform, the transmitted light would be uniform, with the same polarization state across the image. Any lack of uniformity in the orientation of the aragonite tablets that compose the sample would thus manifest in the lack of polarization uniformity of the composite image.

The *P. fucata* cross section had the following dimensions: approximately 2 mm long, approximately 780 µm wide and a thickness that varied linearly along the long dimension with a 4° taper. It was approximately 100 µm at the centre. The cross section was prepared from an intact, vacuum-packed oyster from cnepearls.com. The shell was cut with a diamond saw to create a cross-section sample. The latter was glued to a tripod polisher holder and polished flat. The sample was then removed from the holder, re-glued and subsequently polished with polishing grits down to 0.5 µm at a 4° angle until the tip was on the order of 30–40 µm. The sample was then removed from the holder with acetone and attached to a paperclip for polarimetry and SEM imaging. For the polarimetry experiments, the sample was mounted on a stage that could move in *x-*, *y-* and *z*-directions and tilted up to a 15° angle. SEM images were obtained after polarimetry experiments were completed by coating a portion of the sample with 20 nm of platinum and examining with a JEOL-JSM6360LV SEM in the backscatter mode.

Studies of nacre have indicated that aragonite has its *c*-axis oriented largely parallel to the growth direction [17–20], or normal to the surface of the aragonite tablets. We aligned the sample in our apparatus such that the *c*-axis was along the horizontal component of the incoming light. Thus, in our samples, the *a-* and *b-*axes of the tablets were, barring unforeseen non-uniformities, generally contained in a vertical plane with an unknown orientation. When anti-diagonally polarized light was incident on the sample, it encountered a sample with two indices of refraction: along the horizontal direction, *n_c_* *=* 1.685; and along the vertical direction [31],

2.5 $$n_{ab}={\left(\frac{\text{co}{\text{s}}^{2}\theta }{n_{a}^{2}}+\frac{\text{si}{\text{n}}^{2}\theta }{n_{b}^{2}}\right)}^{-1/2},$$

where *n_a_* = 1.530 and *n_b_* = 1.681, with *θ* being the angle that the *a-*axis forms with the vertical. Thus, the polarization of the transmitted light reflected the phase imparted by the birefringence of the sample. The relative phase between the two components was obtained from the Stokes parameters of equations (2.1)–(2.4) [30]:

2.6 $$\delta =\text{arg}(s_{2}+is_{3}).$$

This phase manifests by changing both the ellipticity and orientation of the polarization ellipse [31]. If the input is anti-diagonal, then when *δ* = 0, the polarization remains linear and anti-diagonal (see legend in figures 3 and 4). As *δ* increases, the polarization becomes left-handed elliptical of decreasing ellipticity, but aligned anti-diagonal, until *δ* = *π*/2 when it becomes left circular. Between *π*/2 and *π* it becomes more left-elliptical again, but oriented diagonally, ending as linearly polarized. Between *π* and 3*π*/2 the polarization becomes right-elliptical of decreasing ellipticity and diagonally oriented, ending as right circular at *δ* = 3*π*/2. Between 3*π*/2 and 2*π* it is right-elliptical, anti-diagonal and with increasing ellipticity.

## 3. Results

**Figure 2.**
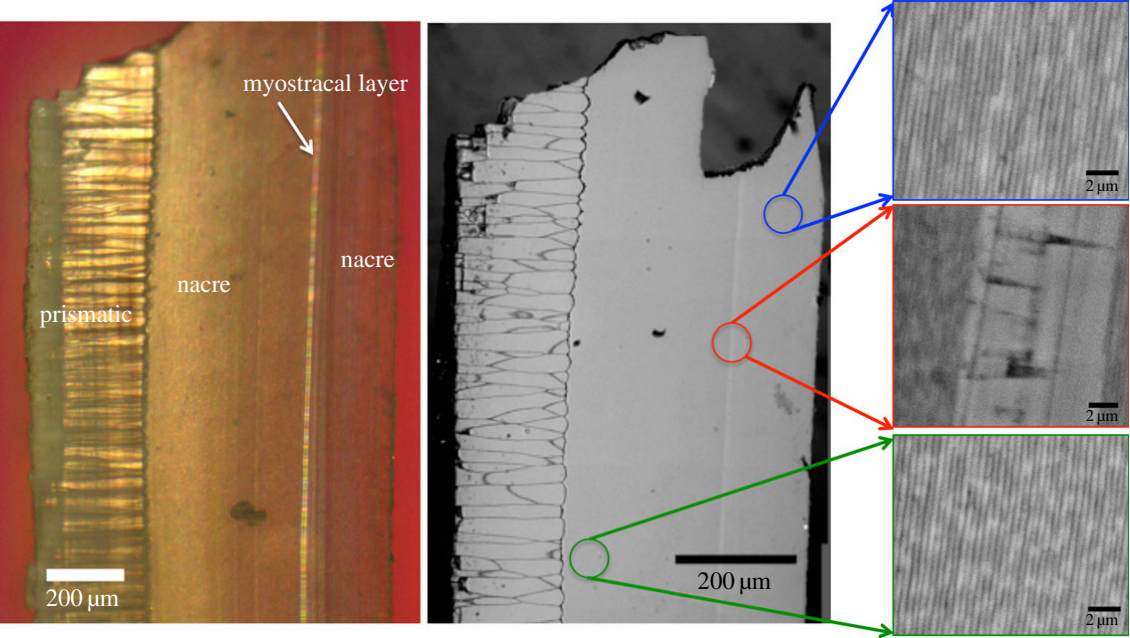
Light microscopy and scanning electron microscopy (SEM) images of a *P. fucata* cross section show the primary structures of interest within the shell, the prismatic layer, nacre and myostracal layer. High-magnification SEM images show the individual structures of the nacre before and after the myostracal layer and the myostracal layer itself. The nacre before and after the myostracal layer is in a brick-and-mortar structure, as illustrated by the lines of tablets (approx. 400 nm thick before the myostracal layer and approx. 500 nm thick after) seen in the SEM images. The myostracal layer appears to consist of solid crystalline blocks.

In figure 2, we present light microscope and SEM images of the *P. fucata* cross section examined with polarimetry. The cross section is oriented such that the prismatic structure consisting of block-like crystals is on the left of the image, followed by nacre on the right. The nacre is split into two distinct regions by a myostracal layer that runs along the length of the cross section and is approximately 10 µm wide. The high magnification SEM images indicate that each of the three regions have distinct structures, with the prismatic region consisting of large block-like crystals that appear to be uniform on the order of 50–100 µm, the nacre consisting of straight lines representing different layers of aragonite tablets, and the myostracal layer consisting of small block-like crystals. As seen within the high-magnification SEM images, the nacre tablets before the myostracal layer are significantly thinner, approximately 400 nm thick, than the nacre tablets that are found after the myostracal layer, approximately 500 nm thick (*p*-value 1 × 10^−13^).

**Figure 3.**
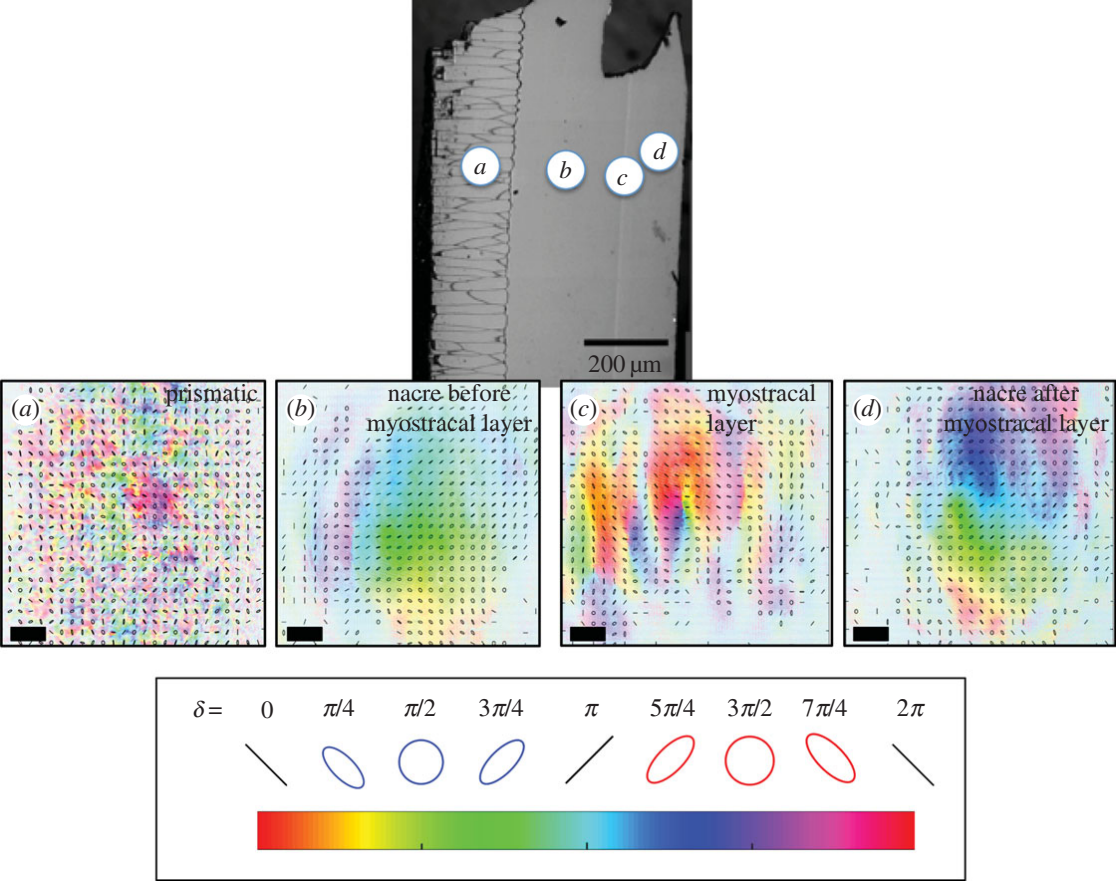
Polarimetry images acquired by sending linearly polarized light, oriented anti-diagonally, through the sample, shown by the SEM image on top. Circles (not to scale) denote the regions corresponding to the polarimetry images below (beam size is approx. 15 µm). The regions are: prismatic (*a*), pre-myostracal nacre (*b*), myostracal layer (*c*) and post-myostracal nacre (*d*). The false colour encodes the phase introduced by the sample onto the horizontal and vertical components of the input polarization. Scale bars of approximately 2 µm in length denote the scale of the polarimetry images. Drawn ellipses at periodic intervals denote the state of polarization at those points. Ellipses oriented either diagonally or anti-diagonally are due to ordered nacre layers with co-aligned *c*-axis and co-planar *a*- and *b*-axes. Other ellipse orientations are due to birefringent sections with crystal axes oriented in other directions.

Figure 3 presents the data observed when linearly polarized, anti-diagonal light was incident on the *P. fucata* cross section of figure 2. Polarimetry allowed us to find the relative phase *δ* imparted by the sample onto the vertical and horizontal components of the incoming light, which we encode in the false colour of the figure, as indicated at the bottom of the figure. We also draw small figures representing the state of polarization at regular intervals, a state that reflects the acquired phase, as mentioned above. Because the sample was cut with a 4-degree wedge, it has a depth that varies in the vertical direction, showing a corresponding change in polarization. We further encoded the intensity of the light in the saturation of the colour.

If the sample had uniform birefringence, corresponding to a single crystallographic orientation, the image would show a phase (colour) that is the same in the horizontal direction, and varying linearly in the vertical direction, with drawn ellipses having semi-major axes aligned either anti-diagonal or diagonal (depending on the value of *δ*) with ellipticity constant in the horizontal direction but smoothly varying in the vertical direction. However, figure 3 indicates that each region of the shell interacts with the incoming light in a unique way. The polarization of the light transmitted through the prismatic layer (A) varies from point to point, as exhibited by all the colours within the image and the overlaid polarization-state figures. The imaged light is essentially randomly polarized.

The light passing through the nacre (B and D) regions, on the other hand, both before and after the myostracal layer shows general uniformity in the polarization. The dependencies of the false colour and the drawn ellipses follow the general expectations of our model: the colours vary smoothly in the vertical direction. Isochrome (same colour) regions are in general horizontal but with important variations that are distinct between the (B) and (D) regions. The myostracal layer (C) results in regular vertical bands of uniform phase that varies in the horizontal direction much more strongly than in the vertical direction. We also observe that the semi-major axes of the state of polarization are no longer diagonal or anti-diagonal, rather we also observe vertical and horizontal, among other orientations. This implies that the axes of the crystals in the myostracal-layer region are aligned differently from those of the nacre regions.

**Figure 4.**
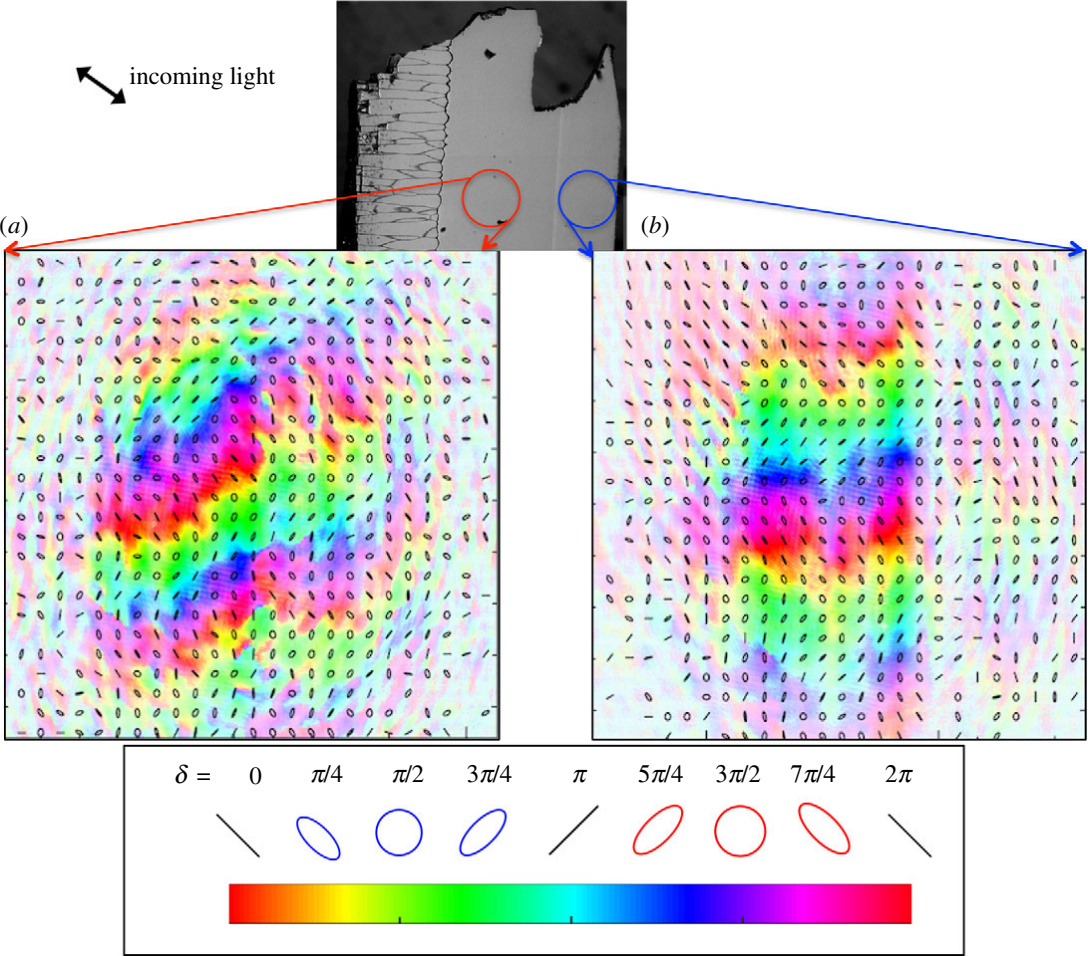
Polarimetry data on the nacre before the myostracal layer (*a*, red) and the nacre after the myostracal layer (*b*, blue) illustrating the structural difference between the two regions. The beam was slightly defocused to a spot size of approximately 150 µm. The false colour encodes the phase introduced by the sample onto the horizontal and vertical components of the input polarization, showing periodic regions or ‘fringes’, with anti-diagonal (red-purple) or diagonal (green-teal) orientations. These reflect the phase imparted by the sample as shown in the scale at the bottom. Drawn ellipses at periodic intervals denote the state of polarization at those points. Before the myostracal layer (*a*), the nacre is shown to have a stepped pattern in which the majority of the transmitted light within this region is linearly polarized. After the myostracal layer (*b*), a similar pattern is observed, though the spacing of the polarization fringes is significantly larger and the fringes appear more regular.

Figure 4 effectively ‘zooms-out’ on the polarization fringes observed in figure 3*b* and *d*. By defocusing the beam, a larger spot size of approximately 150 µm was obtained to provide a larger field of view of the sample, enabling us to better compare the nacre regions on either side of the myostracal layer. Figure 4*a* presents the light transmitted through the nacre located between the prismatic layer and the myostracal layer, while figure 4*b* presents the light transmitted through the nacre at the other side of the myostracal layer. In both regions, the phase imparted onto the transmitted light varies smoothly in the vertical direction. The isochrome regions appear to have a zigzag pattern, with the pattern after the myostracal layer (B) consisting of thicker, more distinct and regular fringes that are generally horizontal in comparison to the pattern before the myostracal layer (A), where the isochrome regions are more jagged and generally inclined with respect to the horizontal. Videos demonstrating how the transmitted light and fringes appear as the sample is moved both vertically and horizontally are available as electronic supplementary material and are consistent with the images in figures 3 and 4 (electronic supplementary material, Video S1 and S2).

## 4. Discussion

The microscopy data of figure 2 show that the myostracal layer consists of block-like crystals that interrupt the nacre structure. The SEM and polarimetry data of figures 2–4 indicate the organization of the nacre formed after the myostracal layer is different from that of the nacre formed before the myostracal layer.

The SEM and visual light microscopy images in figure 2 show that the myostracal layer consists of a completely different structure from that of the nacre on either side of it, with the myostracal layer containing block-like crystals and the nacre, lines of tablets. The SEM images of figure 2 also show that a difference exists between the nacre tablets before and after the myostracal layer, with the tablets located between the myostracal layer and the prismatic layer being significantly thinner (approx. 400 nm thick) than those after the myostracal layer (approx. 500 nm). The polarimetry data of figures 3 and 4 further emphasize that a structural difference exists between the nacre before and after the myostracal layer.

The phase imparted by the sample on the components of the incoming light is determined by

4.1 $$\delta =\frac{2\pi d\Delta n}{\lambda }+\delta_{0},$$

where *d* ≈ 100 µm is the thickness of the sample at the observation point,

4.2 $$\Delta n=n_{c}-n_{ab},$$

λ = 632.8 nm is the wavelength of the light and *δ*_0_ is an overall constant phase. Thus, the measured polarization depends on both the sample thickness and the orientation of the axes of the crystals (*θ*) within the sample, as measured by the index of refraction *n_ab_*. As the sample thickness increases uniformly in the vertical direction due to the wedge shape of the sample, the phase in the vertical direction is a function of both the thickness and orientation. In the horizontal direction, the sample thickness is uniform, thus any phase changes are due to changes in the index of refraction, which in turn is related to the orientation of the crystallites through which the light is transmitted. We can express the change in the phase as

4.3 $$\Delta \delta =\left(\frac{2\pi d}{\lambda }\right)\Delta n_{ab}.$$

The disorder observed in figure 3*a* tells us that the calcium carbonate crystals within the prismatic layer have diverse orientations; in moving from one pixel to the next, vertically or horizontally, approximately 10 µm spatially, we encounter a different phase and polarization, corresponding to distinct types of refraction. Our data align with what others have found in the prismatic layer of *P. fucata*, namely that each individual prism consists of calcium carbonate crystals of multiple orientations, with one prism containing an angular spread of 10–20° [32].

The light transmitted through the myostracal layer, shown in figure 3*c*, also results in strong variation in polarization. Note that the polarimetry image shows vertical fringes of same phase and polarization. Although we have not modelled this feature, it is likely to be due to diffraction caused by the myostracal layer structure itself and distinctly polarized light emerging from the sample at either side of the layer.

The light transmitted through the nacre, both before the myostracal layer, figure 3*b* and figure 4*a*, and after the myostracal layer, figure 3*d* and figure 4*b*, shows significantly less variation in polarization than either the prismatic layer or the myostracal layer. In both nacreous regions the phase changes uniformly in the vertical direction, as evidenced by the pixel colours changing smoothly in line with the colour bar. Equation (4.1) indicates that the phase is dependent upon both orientation and sample thickness: as the sample is wedged, we would expect, with no orientation change, the phase to change uniformly in the vertical direction solely due to sample thickness. As our data indicate a smooth phase change vertically, we attribute the observed phase change to the changing thickness of the sample. Any other information with regard to crystal orientation cannot be extracted from the data in the vertical direction due to unknown overall phase constant, *δ*_0_ (see equation (4.1)). In the horizontal direction, the phase remains fairly constant with slight variations, indicating that most of the nacre tablets are of the same orientation as you move horizontally across the sample, with some having slight differences in the index of refraction or crystallographic orientation. These slight changes in the index of refraction can be calculated via equation (4.3), yielding a variation of the order Δ*n_ab_* = 0.0063 (for Δ*δ* = 2*π* and *d* = 100 µm), which via equation (2.5) represent slight rotations of the aragonite *a-* and *b-*crystallographic axes of about 2.3°.

The lower magnification, shown in figure 4, enables the difference between the nacre before the myostracal layer and the nacre after the myostracal layer to be appreciated. Namely, the horizontal variations in phase observed before the myostracal layer are greater than those after the myostracal layer, with the phase changes appearing diagonal relative to the horizontal, perhaps indicating a continuous rotation of the nacre tablets' *a-* and *b-*axes as they approach the myostracal layer. After the myostracal layer, the phase changes are minimal with slight v-shaped variations that perhaps indicate a set of nacre tablets of different orientation, while the majority of tablets are of the same orientation. In addition, the lower magnification view allows us to see that the phase variations observed in figure 3*b*,*d* occur in repetitive vertical fringes with the fringes before the myostracal layer happening at a higher frequency, being less spaced out, in comparison to the nacre after the myostracal layer. As indicated by equation (4.1), as the vertical thickness changes uniformly on either side of the myostracal layer, any phase change differences between the two sides must be due to changes of the index of refraction. Thus, for this sample, the change in spacing in the fringes indicates that before the myostracal layer there is a greater change in the index of refraction, or crystal orientation, when moving vertically than after the myostracal layer.

Our results highlight structural differences found within the nacre layers of a single shell and raise several interesting questions about whether these structural differences are due to interactions with the myostracal layer. While it is not yet possible to determine whether the difference in nacre organization is caused by the myostracal layer, one could imagine several scenarios in which the myostracal layer influences the chemical or structural nucleation environment for the nacre formed immediately following the myostracal layer, altering the subsequent nacre structure, as observed here. To be able to determine whether such a situation occurs, further research into the relationship between the myostracal layer and the surrounding nacre is needed.

## 5. Conclusion

Through the use of the new technique of polarimetry imaging, we were able to examine the structure of nacre before and after the myostracal layer in *P. fucata*. Before the myostracal layer, the nacre tablets are thinner and largely co-aligned, though appearing to have a continuously varying orientation perhaps representative of slight rotation of the *a-* and *b-*axes about the *c-*axis. After the myostracal layer, the nacre tablets are thicker and more uniform. Further work needs to be done to determine whether the myostracal layer plays a role in ordering the post-myostracal nacre structure.

## Data accessibility

Data supporting this article have been uploaded as part of the electronic supplementary material and deposited at Dryad http://dx.doi.org/10.5061/dryad.dk894 [33].
